# A population-based study on the association between acute renal failure (ARF) and the duration of polypharmacy

**DOI:** 10.1186/1471-2369-13-96

**Published:** 2012-08-30

**Authors:** Yi-Ping Chang, San-Kuei Huang, Ping Tao, Ching-Wen Chien

**Affiliations:** 1 Department of Nephrology, Taoyuan Veterans Hospital, 100 Cheng Kong Rd, Sec. 3, Taoyuan City, 33010, Taiwan; 2 Bureau of National Health Insurance, No.140, Sec.3, Hsinyi Road, Taipei, 10634, Taiwan; 3 Department of Medical Affairs, Taipei Veteran General Hospital, 201 Shihpai Rd. Sec. 2, Taipei, 11217, Taiwan; 4 Institute of Health and Welfare Policy, National Yang Ming University, 155 Linong St., Sec. 2, Beitou, Taipei, 11221, Taiwan; 5 Institute of Hospital and Health Care Administration, National Yang Ming University, 155 Li-Nong St., Sec. 2, Beitou, Taipei, 11221, Taiwan

## Abstract

**Background:**

Because of the rapid growth in elderly population, polypharmacy has become a serious public health issue worldwide. Although acute renal failure (ARF) is one negative consequence of polypharmacy, the association between the duration of polypharmacy and ARF remains unclear. We therefore assessed this association using a population-based database.

**Methods:**

Data were collected from the Taiwan National Health Insurance Research Database (NHIRD) from 2003 through 2006. The case group included patients hospitalized for ARF during 2006, but not admitted due to trauma, surgery, burn trauma, car accident, transplantation, or infectious diseases; the control group included patients hospitalized without ARF. The cumulative number of days of polypharmacy (defined as more than 5 prescriptions per day) for 1 year prior to admission was determined, with patients further subdivided into 4 categories: less than 30 days, 31–90 days, 91–180 days, and over 181 days. The dependent variable was ARF, and the control variables were age, gender, comorbidities in patients hospitalized for ARF, stay in ICUs during ARF hospitalization and site of operation for prior admissions within one month of ARF hospitalization.

**Results:**

Of 20,790 patients who were admitted to hospitals for ARF in 2006, 12,314 (59.23 %) were male and more than 60 % were older than 65 years. Of patients with and without ARF, 16.14 % and 10.61 %, respectively, received polypharmacy for 91–180 days and 50.22 % and 24.12 %, respectively, for over 181 days. A statistical model indicated that, relative to patients who received polypharmacy for less than 30 days, those who received polypharmacy for 31–90, 91–180 and over 181 days had odds ratios of developing ARF of 1.33 (p<0.001), 1.65 (p<0.001) and 1.74 (p<0.001), respectively.

**Conclusions:**

We observed statistically significant associations between the duration of polypharmacy and the occurrence of ARF.

## Background

Polypharmacy has become an emerging public health issue in recent years. Polypharmacy has been correlated with adverse drug reactions (ADRs), increased risk of hospitalization, reduced adherence to medication, and unnecessary expenses [1,2]. In addition, several studies have indicated that polypharmacy is directly or indirectly associated with acute renal failure (ARF) [3-7]. Because the mortality rate of patients hospitalized for ARF is approximately 45 %, and almost 30 % of patients with ARF require renal transplantation [8], further assessments of the association between ARF and polypharmacy would be important in clinical practice.

Methodological problems are present in studies of the relationship between polypharmacy and ARF. First, the definition and measurement of polypharmacy are inconsistent [9-13], making comparisons among studies difficult. For example, polypharmacy has been defined as the concomitant use of five or more drugs, or as the number of unnecessary or inappropriate medications [14-16], with the former definition more widely used by physicians because of its clinical convenience [17]. Second, most previous studies of the association between polypharmacy and ARF have focused only on elderly people, not on the general population [3,5,8,11,14,15,17], thus making the results of these studies difficult to compare across different ages. Third, the involvement of managed care has resulted in fewer patients with polypharmacy, because drug benefit plans of managed care programs vary greatly [17]. Therefore, assessments of the association between polypharmacy and ARF require better health care environments to minimize these interfering factors.

Our aim of this study is to assess the association between the duration of polypharnacy and the development of ARF by analyzing the Taiwan National Health Insurance Research Database (NHIRD). The National Health Insurance (NHI) of Taiwan, first established in 1995, the NHI is a nationwide, compulsory, uniform and comprehensive health insurance system, in which every citizen (beneficiary) is covered by the same health benefits, including a full drug benefit package, excluded over-the-counter (OTC) drugs, in western and Chinese traditional medicine, regardless of the premium paid. Each citizen has access to any doctor or healthcare institution throughout the country, with same and low cost-sharing and/or deductibles. In addition, the NHI has contracts with over 92 % of all clinics and almost 100 % of all hospitals and other healthcare institutions, which have been accredited by standards very similar to those of the Joint Commission International (JCI). The low cost-sharing, similar quality and high accessibility of medical care have resulted in an average of 15.1 outpatient visits per person per year. Each individual receives about 3.35 medications per prescription, much higher than in European countries or the United States. These characteristics make possible a determination of the association between duration of polypharmacy and ARF [18,19]. In addition, the NHIRD contains aggregated utilization records on all individuals throughout the country, including reasons for utilization in ICD-9-CM diagnostic codes and the medication details of each enrollee. This information allowed a determination of the factors influencing ARF, including gender, age, comorbidities, stay in intensive units (ICUs) and major site of operation one month prior to ARF admission, while assessing the association between duration of polypharmacy and ARF.

## Methods

### Database and study design

This retrospective case–control study utilized data obtained from the 2003–2006 NHIRD, which enrolled nearly all 23 million people in Taiwan. The NHIRD, released annually to the public, contains all claim records, including encrypted personal identification number, gender, age, five diagnoses and five treatments(procedure or operation) in ICD-9-CM (International Classification of Disease, Clinical Modification, 9^th^ version) codes, types of lab tests, examinations, medications (in Anatomical Therapeutic Chemical codes), encrypted health institution code, encrypted physician identification and all expenditures for inpatient, outpatient, and emergency department (ED) services. Approval to analyze NHIRD data was obtained from the Ministry of Health, Taiwan (No. 1001460109).

The case group was selected from hospitalized patients diagnosed with ARF (ICD-9-CM: 584) [20-23] for the first time in 2006, with neither inpatient nor outpatient histories of ARF within the previous 3 years; none of these patients had been admitted due to trauma, surgery, burns, car accident, transplant, or infectious diseases. The control group was selected from patients hospitalized in 2006 but without ARF. None of the control patients had an inpatient or outpatient history of ARF within the previous 3 years. Subjects in the control group were matched 1:4 with those in the case group by MDC (major diagnosis category), hospital accreditation level (medical center, regional hospital and community hospital) and month of hospitalization. The case group included 20,790 patients and the control group included 83,160 patients (total, 103,950 patients).

### Study variables

To investigate the association between the duration of polypharmacy and ARF, we retrospectively collected the medication history of all patients for 1 year prior to hospitalization. The duration of polypharmacy was defined as the summation of days a patient took more than 5 prescription medications on a single day in one year. These patients were further subdivided into those who took polypharmacy for less than 30, 31–90, 91–180, and over 181 days. The dependent variable was the occurrence of ARF (yes/no), whereas the control variables were those factors which were found by previous studies to have significant correlation with ARF, they included age, gender, having comorbidities when hospitalized for ARF, stay in ICUs during ARF hospitalization, site of operation for one month prior to ARF admission and number of days taking NSAID(Nonsteroidal anti-inflammatory drugs) or Triamterene. Age was classified into four categories: 0–18, 19–64, 65–79 and over 80 years. A comorbidity was defined as the diagnosis of and treatment with medication(s), as inpatient or outpatient for atherosclerotic heart diseases, congestive heart failure, cerebrovascular accident/transient ischemic attack, peripheral disease, other cardiac disease, chronic obstructive pulmonary disease, gastrointestinal bleeding, liver disease, dysrhythmia, cancer and diabetes; all of these comorbidities have been highly associated with failures in renal functions and have been used often in previous studies [24]. Stay in an ICU was classified as yes or no, depending on whether a patient had stayed in an ICU during the same hospitalization. Site of operation has also been found in previous studies as a factor correlated to ARF hospitalization [25]. In our study, site of operation was defined as having a primary operation (in ICD-9-CM OP codes) on the nervous system, endocrine system, eyes, ears, nose, mouth and pharynx, respiratory system, cardiovascular system, hemic and lymphatic system, digestive system, urinary system, male genital organs, female genital organs, musculoskeletal system, or integumentary system, or as obstetric or miscellaneous diagnostic and therapeutic procedures one month prior to ARF hospitalization. NSAID or Triamterene drugs were also found to be correlated to renal failure [26,27]. Therefore, we also included number of days taking both classes of drugs as a control variable.

### Statistical analysis

Data were analyzed using SAS version 9.2 (SAS Institute Inc., Cary, NC, USA). In the analysis of single dependent variables, we first used descriptive statistics to determine the distribution of age, gender, comorbidities, ICU and site of operation in patients with and without ARF and in patients with cumulative use of polypharmacy for less than 30, 31–90, 91–180, and over 181 days. *χ*^2^ and *t* tests were used to assess the association between the duration of polypharmacy and the occurrence of ARF and to investigate the variables that significantly affected ARF. Stepwise logistic regression analysis was utilized to determine the association between ARF and the duration of polypharmacy after controlling for patient variables.

## Results

The demographic and clinical characteristics of the case and control groups are shown in Table 1. We found that ARF was significantly correlated with male gender (p<0.001), with 59.23 % of patients with ARF being male. ARF also increased with age; of ARF patients, 0.84 % were aged 0–18 years, 31.78 % were aged 19–64 years, 38.63 % were aged 65–79 years and 28.75 % were over 80 years old. When the study samples were paired with hospital accreditation status, the results of χ^2^ tests were not significant. When we assessed the association between ARF and duration of polypharmacy, we found that a higher proportion of case than control patients received polypharmacy for 91–180 (16.14 % vs. 10.61 %) and for over 181 days (50.22 % vs. 24.12 %).

**Table 1:** Descriptive statistics of subjects in case and control groups

	**Control Group (N = 83,160)**		**Case Group (N = 20,790)**		**Crude odds ratio**	**p-value**
	**Number**	**(%)**	**Number**	**(%)**		
Gender						<0.001
Male	44,695	(53.75)	12,314	(59.23)	1.25	
Female(ref)	38,465	(46.25)	8,476	(40.77)		
Age						<0.001
0-18 years old (ref)	15,065	(18.12)	174	(0.84)		
19-64 years old	39,199	(47.14)	6,607	(31.78)	14.57	
65-79 years old	19,684	(23.67)	8,032	(38.63)	35.28	
Over 80 years old	9,212	(11.08)	5,977	(28.75)	56.10	
Hospital accreditation status						N.A
Medical center	33,544	(40.34)	8,386	(40.34)		
Regional hospital	35,240	(42.38)	8,810	(42.38)		
Community hospital	14,376	(17.29)	3,594	(17.29)		
Duration of polypharmacy						<0.001
Less than 30 days(ref)	40,408	(48.59)	4,069	(19.57)		
31-90 days	13,867	(16.68)	2,925	(14.07)	2.09	
91-180 days	8,823	(10.61)	3,355	(16.14)	3.78	
Over 181 days	20,062	(24.12)	10,441	(50.22)	5.17	
ICU						<0.001
Yes	9,454	(11.37)	9,768	(46.98)	6.91	
NSAID_S_ or Triamterene day*	4.78	(17.23)	8.10	(22.61)	1.01	<0.001
Comorbidity**						
ASHD	12,387	(14.90)	5,730	(27.56)	2.17	<0.001
CHF	5,876	(7.07)	4,711	(22.66)	3.85	<0.001
CVA/TIA	9,181	(11.04)	5,289	(25.44)	2.75	<0.001
PVD	2,946	(3.54)	1,578	(7.59)	2.24	<0.001
Other cardiac	5,641	(6.78)	2,538	(12.21)	1.91	<0.001
COPD	14,477	(17.41)	5,605	(26.96)	1.75	<0.001
GI	14,191	(17.06)	6,788	(32.65)	2.36	<0.001
Liver disease	10,338	(12.43)	3,998	(19.23)	1.68	<0.001
Dysrhythmia	5,783	(6.95)	3,952	(14.20)	2.21	<0.001
Cancer	7,718	(9.28)	3,989	(19.19)	2.32	<0.001
Diabetes	16,390	(19.71)	8,396	(40.38)	2.76	<0.001
Site of operation						
Nervous system	1,564	(1.88)	285	(1.37)	0.73	<0.001
Endocrine system	1,168	(1.40)	16	(0.08)	0.05	<0.001
Eyes	178	(0.21)	42	(0.20)	0.95	0.736
Ears	79	(0.09)	6	(0.03)	0.30	0.003
Nose, mouth and pharynx	668	(0.80)	67	(0.32)	0.40	<0.001
Respiratory system	4,023	(4.84)	1,902	(9.15)	1.98	<0.001
Cardiovascular system	7,465	(8.89)	5,165	(24.84)	3.35	<0.001
Hemic and lymphatic system	841	(1.01)	265	(1.27)	1.26	0.001
Digestive system	11,578	(13.92)	4,471	(21.51)	1.69	<0.001
Urinary system	8,805	(10.59)	1,286	(6.19)	0.56	<0.001
Male genital organs	71	(0.09)	4	(0.02)	0.23	0.002
Female genital organs	164	(0.20)	1	(0.00)	0.03	<0.001
Obstetrical procedures	0	(0.00)	0	(0.00)	-	-
Musculoskeletal system	2,933	(3.53)	508	(2.44)	0.69	<0.001
Integumentary system	2,218	(2.67)	626	(3.01)	1.13	0.007
Miscellaneous diagnostic and therapeutic procedures	27,814	(33.45)	12,863	(61.87)	3.23	<0.001

* *t* test.

**Abbreviations: ASHD, atherosclerotic heart disease; CHF, congestive heart failure;COPD, chronic obstructive pulmonary disease; CVA/TIA, cerebrovascular accident/transient ischemic attack; GI, gastrointestinal bleeding; PVD, peripheral vascular disease.

Statistical analyses showed that all comorbidities tested were significantly correlated with ARF (p<0.001), with all odds ratios being >1.00. A higher percentage of patients in the case group stayed in the ICU during hospitalization (46.98 % vs. 11.37 %). Site of operation was significantly correlated with ARF (p<0.001), except for operations on the ears (p<0.736). The case group had significantly longer days in taking NSAID or Triamterene drugs than the control group (8.10 ± 22.61 days vs. 4.78 ± 17.23 days, p<0.001).

Table 2 shows the results of a stepwise logistic regression analysis, in which ARF was the dependent variable, polypharmacy duration was the independent variable, and the influence of gender, age, ICU, comorbidities and site of operation were controlled. Compared with cases who received polypharmacy for less than 30 days, all other polypharmacy categories were at higher risks for ARF (p<0.001), with the odds ratios (OR) increasing with increasing duration of polypharmacy, being 1.33, 1.65 and 1.74, in patients who received polypharmacy for 31–90, 91–180, and over 181 days, respectively. Males were 1.32 (p<0.001) times more likely than females to develop ARF and increased age was also associated with ARF. Relative to patients aged 0–18 years old, those aged 19–65, 65–79, and over 80 years had ORs for the development of ARF of 6.40, 8.93 and 14.46, respectively. Stay in the ICU was also significantly associated with the development of ARF (OR 4.37; p<0.001).

**Table 2:** Stepwise logistic regression of the duration of polypharmacy and ARF

	$\hat{\beta }$	**SE(**$\hat{\beta }$**)**	**Adjusted odds ratio**	**p-value**
Duration of polypharmacy: Less than 30 days*				
31- 90 days	0.28	0.03	1.33	<0.001
91 – 180 days	0.50	0.03	1.65	<0.001
Over 181 days	0.55	0.03	1.74	<0.001
**Gender**:Female*				
Male	0.28	0.02	1.32	<0.001
**Age:**0-18 years old *				
19-64 years old	1.86	0.08	6.40	<0.001
65-79 years old	2.19	0.08	8.93	<0.001
Over 80 years old	2.67	0.08	14.46	<0.001
ICUs	1.48	0.02	4.37	<0.001
Comorbidity				
ASHD	−0.23	0.02	0.80	<0.001
CHF	0.67	0.03	1.95	<0.001
CVA/TIA	0.21	0.02	1.24	<0.001
PVD	0.14	0.04	1.15	0.001
Other cardiac	0.08	0.03	1.09	0.006
COPD	−0.10	0.02	0.91	<0.001
GI	0.30	0.02	1.36	<0.001
Liver disease	0.15	0.02	1.16	<0.001
Cancer	0.65	0.02	1.92	<0.001
Diabetes	0.46	0.02	1.58	<0.001
Site of operation				
Nervous system	−0.82	0.07	0.44	<0.001
Endocrine system	−2.25	0.26	0.11	<0.001
Nose, mouth and pharynx	−0.58	0.14	0.56	<0.001
Respiratory system	−0.30	0.03	0.75	<0.001
Cardiovascular system	0.38	0.02	1.47	<0.001
Digestive system	0.26	0.02	1.30	<0.001
Urinary system	−0.21	0.03	0.81	<0.001
Male genital organs	−1.38	0.54	0.25	0.010
Female genital organs	−2.63	1.01	0.07	0.009
Musculoskeletal system	−0.15	0.06	0.86	0.007
Integumentary system	0.11	0.05	1.12	0.039
Miscellaneous diagnostic and therapeutic procedures	0.35	0.02	1.42	<0.001

* reference group.

All comorbidities, except for atherosclerotic heart disease, dysarrhythmia and chronic obstructive pulmonary disease, were associated with higher risks of ARF (OR >1.00 each; p<0.001). Only surgical operations on the cardiovascular system, digestive system, integumentary system and miscellaneous diagnostic and therapeutic procedures showed ORs >1.00 and with statistical significant (p<0.05 each). In contrast, operations on the nervous system, endocrine system, nose, mouth and pharynx, respiratory system, urinary system, male genital organs, female genital organs and musculoskeletal system had a negative coefficient (between −2.63 and −0.15) compared with other sites of operation. Number of NSAID or Triamterene days was not selected to be included in the stepwise logistic regression model.

## Discussion

Our analysis of a dataset from the population-based NHIRD revealed a positive association between the duration of polypharmacy and the occurrence of ARF. Even after controlling for other factors, a longer duration of polypharmacy was associated with a higher risk of ARF.

The adoption of managed care systems by more countries has made research focusing on polypharmacy [17,28], especially duration of polypharmacy, more difficult to perform. Usually the medications prescribed to enrollees in managed care programs are highly controlled, whereas non-prescription medications are not. The lack of information about non-prescription medications makes it very difficult to trace polypharmacy under managed care environments. The actual occurrence of polypharmacy may be higher than that reported, since many people do not inform their doctors about taking non-prescription drugs regularly. This situation, however, is rare in Taiwan, because the NHI pays for most drugs for acute and chronic diseases, and enrollees seldom spend their own money to buy nonprescription drugs regularly. It has made it easier to assess polypharmacy in Taiwan than in other countries. Moreover, since we defined polypharmacy as the concomitant use of more than five medications and we assessed the cumulative days of polypharmacy, we were better able to assess the duration of polypharmacy and the association of duration with ARF (or adverse drug events leading to ARF) than previous studies, which assessed only the correlation between polypharmacy and ARF [1,2,4,7,10,29-31]. Indeed, we found that patients with a longer duration of polypharmacy were at higher risk for ARF hospitalization, consistent with previous findings reporting that excessive medication intake can cause renal damage and ARF [32-34]. Although we did not observe any significant differences between our case and control groups in the reasons for medication, we found that the average duration of polypharmacy was longer in the case than in the control group (191 days vs. 146 days). Therefore, the main cause of ARF may not be the medication itself, but rather the cumulative effects of polypharmacy. After controlling for confounding factors, including comorbidities, stay in the ICU during ARF hospitalization or site of operation within one month prior to ARF hospitalization, we still found that the risk of ARF was associated with duration of polypharmacy.

Consistent with previous reports, we found that polypharmacy was more likely to occur in elderly people [3,9,12]. The cumulative duration of polypharmacy in elderly people was likely higher because many of these individuals may take more than 5 medications per day due to multiple chronic diseases, including gastrointestinal bleeding, diabetes, chronic obstructive pulmonary disease and atherosclerotic heart disease. The optimal strategy to prevent ARF in elderly people may be to encourage doctors to avoid prescribing multiple medications or medications with renal toxicity to elderly people, or to prescribe medications at minimal doses [35]. This, however, may become more unrealistic since the elderly population in many countries is growing rapidly. Therefore, more research is necessary to reduce inappropriate medications in elderly patients requiring long duration of polypharmacy.

We also found that comorbidities, ICU stay during hospitalization for ARF and site of operation within one month prior to ARF hospitalization were associated with higher risks of ARF, findings consistent with previous reports [36,37]. Our finding, however, that dysrhythmia was not associated with a risk of ARF, differed from those showing associations between cardiac diseases and ARF [38-40]. Our result may have been because of the inclusion of too many cardiac comorbidities in our logistic regression analysis. Although the number of days taking NSAID or Triamterene drugs was found significantly different between two groups, it was not selected in stepwise logistic regression model. It was probably because the number of days taking NSAID or triamterene drug was highly correlated with the duration of polyphamacy.

This study had two main limitations. First, we relied on medication claims in the NHIRD database to define polypharmacy. In reality, some patients may have purchased or taken medications on their own, thus underestimating the actual occurrence and duration of polypharmacy. However, due to the high accessibility of Taiwan’s universal health insurance and the wide coverage for medications, few people would purchase medications on their own. Therefore, the influence of this underestimation on our results were likely minimal. Second, this study was limited by the use of this database, in that our case group included patients without a history of ARF for the previous three years, minimizing the effects of previous ARF on current ARF.

## Conclusions

We observed statistically significant associations between the duration of polypharmacy and the occurrence of ARF in this study. However, this association needs further study to ensure a causal relationship. In addition, the study of duration of polypharmacy also needs further attentions to specific group of people, such as elderly patients with multiple chronic diseases, since the society of many developed countries age very soon.

## Competing interests

The authors declare that they have no competing interests.

## Authors’ contributions

YPC and CWC designed the research protocol; PT performed the literature searches; SKH and CWC analyzed the data; and YPC and CWC wrote the paper. All authors read and approved the final manuscript.

## Pre-publication history

The pre-publication history for this paper can be accessed here:

http://www.biomedcentral.com/1471-2369/13/96/prepub
